# Supplemental macronutrients and microbial fermentation products improve the uptake and transport of foliar applied zinc in sunflower (*Helianthus annuus* L.) plants. Studies utilizing micro X-ray florescence

**DOI:** 10.3389/fpls.2014.00808

**Published:** 2015-01-21

**Authors:** Shengke Tian, Lingli Lu, Ruohan Xie, Minzhe Zhang, Judith A. Jernstedt, Dandi Hou, Cliff Ramsier, Patrick H. Brown

**Affiliations:** ^1^MOE Key Laboratory of Environment Remediation and Ecological Health, College of Environmental and Resource Science, Zhejiang UniversityHangzhou, China; ^2^Department of Plant Sciences, University of CaliforniaDavis, Davis, CA, USA; ^3^Ag Spectrum CompanyVero Beach, FL, USA

**Keywords:** foliar fertilizer, remobilization, sunflower, μ-XRF, zinc, microbial fermentation, biostimulant

## Abstract

Enhancing nutrient uptake and the subsequent elemental transport from the sites of application to sites of utilization is of great importance to the science and practical field application of foliar fertilizers. The aim of this study was to investigate the mobility of various foliar applied zinc (Zn) formulations in sunflower (*Helianthus annuus* L.) and to evaluate the effects of the addition of an organic biostimulant on phloem loading and elemental mobility. This was achieved by application of foliar formulations to the blade of sunflower (*H. annuus* L.) and high-resolution elemental imaging with micro X-ray fluorescence (μ-XRF) to visualize Zn within the vascular system of the leaf petiole. Although no significant increase of total Zn in petioles was determined by inductively-coupled plasma mass-spectrometer, μ-XRF elemental imaging showed a clear enrichment of Zn in the vascular tissues within the sunflower petioles treated with foliar fertilizers containing Zn. The concentration of Zn in the vascular of sunflower petioles was increased when Zn was applied with other microelements with EDTA (commercial product Kick-Off) as compared with an equimolar concentration of ZnSO_4_ alone. The addition of macronutrients N, P, K (commercial product CleanStart) to the Kick-Off Zn fertilizer, further increased vascular system Zn concentrations while the addition of the microbially derived organic biostimulant “GroZyme” resulted in a remarkable enhancement of Zn concentrations in the petiole vascular system. The study provides direct visualized evidence for phloem transport of foliar applied Zn out of sites of application in plants by using μ-XRF technique, and suggests that the formulation of the foliar applied Zn and the addition of the organic biostimulant GroZyme increases the mobility of Zn following its absorption by the leaf of sunflower.

## INTRODUCTION

Sunflower (*Helianthus annuus* L.) is an oilseed crop of great importance worldwide, due to the excellent quality of the oil extracted from its seeds that are consumed in various ways. Cultivation of the sunflower is becoming increasingly significant globally (Coelho Bezerra et al., 2014). In 2008–2009, the world sunflower seed production was about 33 million tones, around 8.5% of the world’s total oilseed production (), the leading producers of which are the EU, Russia, Ukraine, Argentina, USA, China, India, and Turkey (Lomascolo et al., 2012).

Foliar fertilization is an increasingly popular practice with particular importance for the production of high value crops (Fernández and Brown, 2013; Fernández et al., 2013) such as sunflowers with many examples of positive responses to foliar application of micronutrients, including zinc (Zn), iron (Fe), boron (B), manganese (Mn), and molybdenum (Mo), on the seed yield and seed quality of sunflower (Jabeen et al., 2013; Skarpa, 2013; Skarpa et al., 2013; Tohidi-Moghadam, 2013; Yang et al., 2013; Tahir et al., 2014). Foliar fertilization has particular value in overcoming nutrient deficit resulting from stress conditions, such as salinity and drought, which often compromise root growth and decrease root absorption capacity (Kannan, 2010). Foliar application of Zn resulted in a greater improvement in Zn densities in rice and wheat grain when compared with soil applied Zn (Ebrahimian et al., 2010; Phattarakul et al., 2012; Zhang et al., 2012).

Foliar fertilization is theoretically more immediate and target-oriented than soil fertilization since nutrients can be directly delivered to plant tissues during critical stages of plant growth (Fernández and Brown, 2013; Fernández et al., 2013). Optimizing the efficacy of the foliar applied nutrients is therefore of great importance from an economic, agronomic and environmental point of view. Our understanding of the factors that determine the ultimate efficacy of foliar applications remains poor and the response of plants to foliar Zn applications is highly variable (Fernández and Brown, 2013). Many reports indicate that foliar application of Zn may significantly increase the concentrations of Zn in the applied leaves but may have little effect on foliar concentrations in non-sprayed tissues or tissue that develop subsequent to the foliar application (Zhang and Brown, 1999; Mirzapour and Khoshgoftar, 2006; Sanchez et al., 2006). The factors that control the uptake and subsequent translocation of foliar applied nutrients out of the leaf, and the effect of spray formulation on these processes are poorly understood (Bukovac, 1985; Adamec, 2013; Fernández and Brown, 2013). While various approaches have been used to determine the efficacy of foliar applied nutrients using stable and radioactive isotopic labeling (Wittwer and Teubner, 1959; Bukovac, 1985; Sah and Brown, 1998; Boaretto et al., 2001; Sanchez et al., 2006), it remains challenging to determine the pathways of mobilization from leaf to shoot and to monitor the influence of foliar formulation on phloem loading and micronutrient transport.

To address the inherently low efficiency of many foliar Zn formulations, a wide range of commercial products have been developed and marketed (Fernández et al., 2013). Recently, there has been much interest in the incorporation of organic molecules (sugars, amino acids, polysaccharides etc.) or biostimulants into foliar fertilizers with the rationalization that these additives will enhance the uptake, or subsequent mobility of the applied nutrient (Fernández et al., 2013). The term ‘biostimulant’ is used to describe a substance or material, with the exception of nutrients and pesticides, which when applied to plants has the capacity to beneficially modify plant growth (Calvo et al., 2014). Currently there is very little scientific evidence that biostimulants can specifically enhance the uptake and utilization of foliar applied fertilizer materials.

The technique of X-ray fluorescence (XRF) has been widely used in the research of elemental distribution in plant tissues, and has proved to be a promising tool to study *in vivo* localization of metals in plants due to its high-resolution and sensitivity (Punshon et al., 2009; Zhao et al., 2014). XRF analyses can be performed to visualize cellular and subcellular distribution of elements in plants without significant pretreatment of the samples. We have previously applied this technique to characterize the location and to monitor changes in concentration and distribution of Zn during plant development or following foliar applications (Lu et al., 2013a,b; Tian et al., 2014). In this current study, we will utilize μ-XRF to obtain high spatial quantification (cellular and subcellular) of elemental distribution and transport following the application of various Zn formulations with the aim of: (1) increasing our understanding of the processes that govern the localization and transport of foliar applied nutrients with emphasis on Zn, and (2) to determine if the formulation of the foliar applied Zn, with addition of macronutrients or biostimulant, alters the mobility of the element following its absorption by the leaf of sunflower.

## MATERIALS AND METHODS

### PLANT CULTURE

Sunflower (*H. annuus* L.) seeds were imbibed at 4^∘^C for 2 days and allowed to germinate in darkness at room temperature for 3 days. Seedlings were planted in potting soil. The pots were in 20 cm diameter, 20 cm tall, and filled with a soil mixture containing (% of volume) 40% peat, 35% silica clay, 20% perlite, and 5% gravel. One plant per pot, and placed in a greenhouse (photo flux density of 400 μmol m^-2^s^-1^, light/dark period of 16/8 h, day/night temperature of 25/20^∘^C and day/night humidity of 70/85%). Plants were watered as needed by irrigation (usually twice per day) with a nutrient solution of the following composition: 1.2 mM KNO_3_, 0.8 mM Ca(NO_3_)_2_, 0.8 mM NH_4_NO_3_, 0.3 mM KH_2_PO_4_, and 0.2 mM MgSO_4_, 12 μM Fe-EDTA, 0.25 μM Na_2_B_8_O_13_⋅4H_2_O, 1.5 μM MnSO_4_, 0.25 μM ZnSO_4_, 0.5 μM CuSO_4_, 0.04 μM Na_2_MoO_4_.

### TREATMENTS WITH DIFFERENT FOLIAR FERTILIZERS

After 1 month of growth, plants of sunflowers were watered with nutrient solution without Zn for 7 days. Fully expanded leaves of sunflowers were then washed thoroughly with deionized water, and immersed for 10 s in 250 mL solution containing different nutrients. Treatments include surfactant sprayed control, “CleanStart,” “Kick-Off,” “CleanStart” + “Kick-Off,” “CleanStart” + “Kick-Off” + “GroZyme,” and ZnSO_4_. All materials were applied so that final Zn concentrations in the applied material and volume of applied materials were equivalent. The nutrient composition of the different treatments is shown in **Table 1**. Because of the complex nature of the commercial products used, it was not possible to prepare a control spray treatment that contained equivalent amounts of all nutrient elements present in Kick-Off or CleanStart. At the rate used here, GroZyme contains negligible concentrations of all essential plant elements. To avoid the possibility that the effect of the foliar spray was a consequence of alleviation of secondary (not Zn) nutrient deficiency, all plants were grown with continuous and abundant soil nutrient. Leaf analysis was conducted and all nutrients were found to be present at adequate levels and plants showed no sign of nutrient deficiency. The foliar fertilizer product “CleanStart,” “Kick-Off,” and “GroZyme” were obtained from Ag Spectrum Company (DeWitt, IA, USA). 0.1% Silwet L-77 (v/v) was added in each solution. The solutions were applied to the leaves of sunflower 8 h before darkness (10 am), and all plant tissues except the sprayed leaf were covered to prevent inadvertent spray application. The petioles of all sprayed leaves were carefully protected by coating the leaf base petiole junction with lanolin (Sigma) and Teflon membranes. Four plants were treated as one replication, with three replications for each treatment. The foliar application of the fertilizers was replicated one time after 7 days, and then plants were harvested 7 days later.

**Table 1:** **Elemental composition of the foliar fertilizers (mg L^–1^ in solution)**.

Treatments	Control	CleanStart	Kick-Off	Kick-Off + CleanStart	Kick-Off + CleanStart + GroZyme	ZnSO_4_
N	–	3200	–	3200	3200	–
P_ 2_O_5_	–	7600	–	7600	7600	–
K_ 2_O	–	1200	–	1200	1200	–
S	–	–	200	200	200	–
Co	–	–	5	5	5	–
Cu	–	–	50	50	50	–
Fe	–	–	100	100	100	–
Mo	–	–	100	100	100	–
Mn	–	–	5	5	5	5
Zn	–	–	200	200	200	200

GroZyme is a microbial fermentation product derived from a proprietary mix of organic cereal grains inoculated with specific bacterial cultures and fermented. The fermentation process occurs under controlled environmental conditions until a specific metabolic profile is achieved at which time the live bacterium are liaised and the material is filtered to remove large particles. This concentrate is then extended and stabilized to make the final product. Soil applications of GroZyme have been reported to alter soil microbial activity and nitrogen transformations (Chen et al., 2002, 2003). The metabolic basis for the biological activity of foliar applied GroZyme is not known. However, field observations suggest that GroZyme functions to enhance plant growth by enhancing K metabolism and sugar transport (Ag Spectrum, unpublished results). CleanStart is derived from ammonia, urea, orthophosphoric acid and potassium hydroxide, and Kick-Off is a micronutrient mix of Fe, Mn, Cu, Zn predominantly derived from nitrate sources with additional surfactants and stabilizers. The elemental composition of all spray applications is provided in **Table 1**.

### ELEMENTAL ANALYSIS

The petioles of leaves were oven-dried at 65^∘^C for 72 h, then ground using a stainless steel mill and passed through a 0.25-mm sieve for analysis of nutrient elements. Ground, dry plant samples (0.1 g) of each treatment were digested with 5.0 mL HNO_3_-HClO_4_ (v/v: 4:1), and the digest was transferred to a 50-mL volumetric flask, made up to volume with water and filtered. Concentrations of mineral elements (i.e., Zn, Fe, Cu, Mn, B, K, Ca, and Mg) in the filtrates were analysed using inductively coupled plasma mass spectroscopy (ICP-MS; Agilent 7500a, USA).

### ELEMENTAL MAPPING BY μ-XRF

#### Sample preparation

Mid-sections of leaf petioles were cut from the leaves treated with different foliar treatments. Leaf cross-sections (100 μm thick) were cut with a cryotome (LEICA, CM1850) at a temperature of -20^∘^C (Tian et al., 2014). Single sections of each treatment were selected under light microscopy for their ultrastructural integrity and then freeze-dried under -20^∘^C for 3 days prior to μ-XRF analysis. Since μ-XRF analysis is time consuming and expensive only single samples from each treatment could be analyzed. Given that true replicate analyses could not be performed additional steps were taken to avoid the potential for experimental artifacts and to avoid any sample selection or analysis bias. All treatments were carefully controlled such that treatment conditions and experimental duration were identical; petioles were then taken from the four replicate plants and multiple sections from each petiole were prepared as described above. All sections were then assessed by light microscopy for ultrastructural integrity and a single section was then selected and transported to the Stanford Synchrotron Radiation Laboratory (SSRL) for μ-XRF analysis. Samples selected in this fashion, therefore represent unbiased examples of treatment effects.

#### μ-XRF analysis

Micro-XRF imaging was performed on the SSRL using beamlines 2–3. Experiments on SSRL beamline 2–3 were recorded at 13 500 eV. The incident X-ray beam of 2 μm in beamline 2–3 was focused using a pair of Kirkpatrick-Baez mirrors, and the incident beam was monochromatized using a Si (111) double-crystal monochromator. Micro-XRF maps were obtained by rastering the beam at 20 and 5 μm steps, with a count time of 200 ms per step, for the following major and minor/trace elements: P, S, Cl, K, Ca, Mn, Fe, Cu, and Zn. Fluorescence signal intensities for the above elements were calculated in SMAK software (Webb, 2014). The fluorescence data were presented as tricolor maps that allow for the spatial distribution of three elements to be shown. Pixel brightness was displayed in RGB, with the brightest spots corresponding to the highest element fluorescence.

### STATISTICAL ANALYSIS OF DATA

All data were statistically analyzed using SPSS (Version 12.0). The figures were made using the software Origin 8.0.

## RESULTS

### DISTRIBUTION PATTERNS OF NUTRIENTS IN LEAF VEIN

**FIGURE 1:**
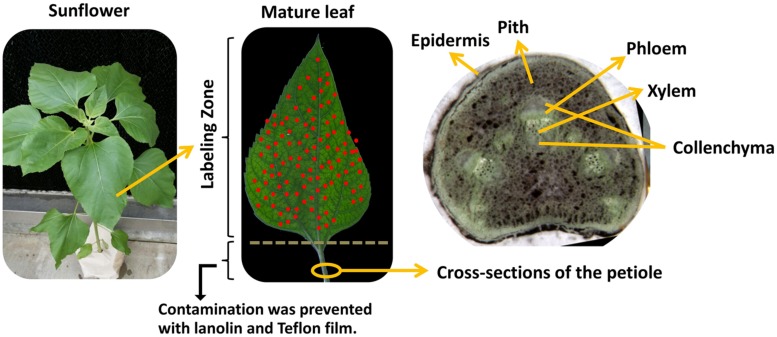
**Microscope image of petiole cross-section collected from sunflowers plants after foliar application.** Fully expanded leaves of 1-month old sunflower were treated with different foliar fertilizers, then cross-sections of the petiole were cut by cryotome at -20^∘^C 7 days after final foliar fertilization treatment.

To investigate the effects of different fertilizers on retranslocation of nutrients in the leaves of sunflower, μ-XRF mapping was performed. Cross sections of petioles were cut from the sunflower plants at approximately 1.0 cm below the leaves and imaged under a light microscope prior to utilization for μ-XRF imaging (**Figure 1**). The cross section of petiole was composed of epidermis, parenchyma, vascular bundle containing xylem, phloem, and surrounding collenchyma. The microscope image in **Figure 1** shows that the phloem within the vascular bundle exists as a discrete layer of cells on the abaxial (morphological bottom) side of the vascular bundle with xylem on the adaxial or upper side of the petiole. The entire vascular bundle is enclosed in tissue that is likely collenchyma.

**FIGURE 2:**
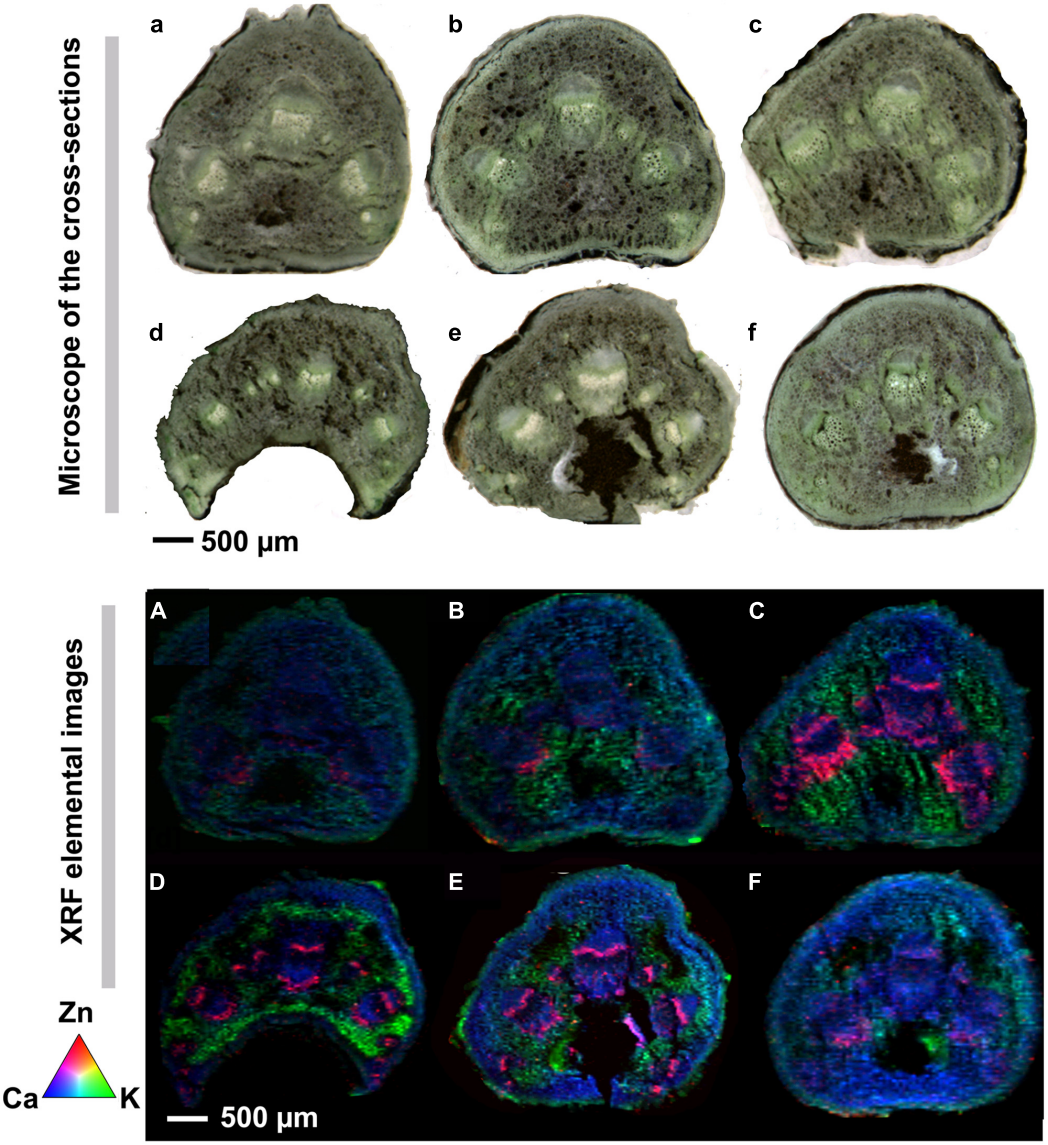
**Microscope cross sections **(a–f)** and μ-XRF elemental maps **(A–F)** for Zn (red), Ca (green), and K (blue) of petioles collected from sunflowers subject to different foliar fertilizers.** Cross-sections of leaf veins were cut from sunflower plants treated with **(a, A)** control, **(b, B)** CleanStart, **(c, C)** Kick-Off, **(d, D)** CleanStart + Kick-Off, **(e, E)** CleanStart + Kick-Off + GroZyme, and **(f, F)** ZnSO_4_, and then analyzed by μ-XRF imaging. Compositions of the nutrients in different treatments are shown in **Table 1**. Pixel brightness for μ-XRF map **(A–F)** is displayed in RGB, with the brightest spots corresponding to the highest element fluorescence. Scale bar: 500 μm.

Integrated intensity for Zn and other elements were calculated from the spectrum and normalized by the intensity of the Compton scattering peak. Elemental mapping for the measurement area was obtained from the normalized intensity for each element. The elemental distribution maps of Zn (red), Ca (blue), and K (green) in the petioles collected from sunflowers plants with different treatments are presented in **Figures 2A-F**, together with corresponding photographs taken using an optical microscope (**Figures 2a-f**). The quantification of the fluorescence yields was normalized by I_0_ and the dwell time. The normalized X-ray fluorescence intensities were scaled to different color brightness for individual elements, with the brightest spots corresponding to the highest elemental fluorescence. Each map indicates the relative distribution of the three elements, and the scale of fluorescence counts for individual elements is the same for each map.

Very slight signals of Zn (red color) was noted in the petioles collected from the control and CleanStart treated plants of sunflower. The greatest concentration of Zn evident in the collenchyma immediately adjacent to the xylem (**Figures 2a,b,A,B**). At the resolution used in these experiments it is not possible to determine if the deposition of Zn in regions other than the phloem was resent in xylem or in collenchyma immediately adjacent to the xylem (referred to here as xylem/collenchyma). A modest increase in the K signal in petioles was also observed in the CleanStart treatment. Foliar application of “Kick-Off,” a product containing Co, S, Fe, Cu, Mn, Mo, and Zn chelated with EDTA (**Table 1**) to sunflower leaves resulted in a marked increase in the concentration of Zn detected in the petioles with a notable deposition in a narrow band corresponding to the phloem tissues of the petiole and a more diffuse band in the xylem/collenchyma region (**Figures 2c,C**), while no such preferential localization to phloem tissues was noted for control, CleanStart or ZnSO_4_ treatments and no phloem specific accumulation of other elements was observed (data not shown). Application of ZnSO_4_ at the same Zn levels (200 mg L^-1^) as used for all Zn treatments to the sunflower leaves also increased phloem Zn in the petioles (**Figure 2F**) as compared with the controls (**Figure 2A**), but the effect is much less pronounced than that of “Kick-Off.” The combined foliar application of “Kick-Off” with “CleanStart” resulted in a similar enhancement in Zn uptake and preferential distribution of Zn to phloem tissues and xylem/collenchyma. The addition of the biostimulant product GroZyme resulted in a much more concentrated enrichment of Zn in the phloem and xylem/collenchyma region of the petiole vascular bundle.

**FIGURE 3:**
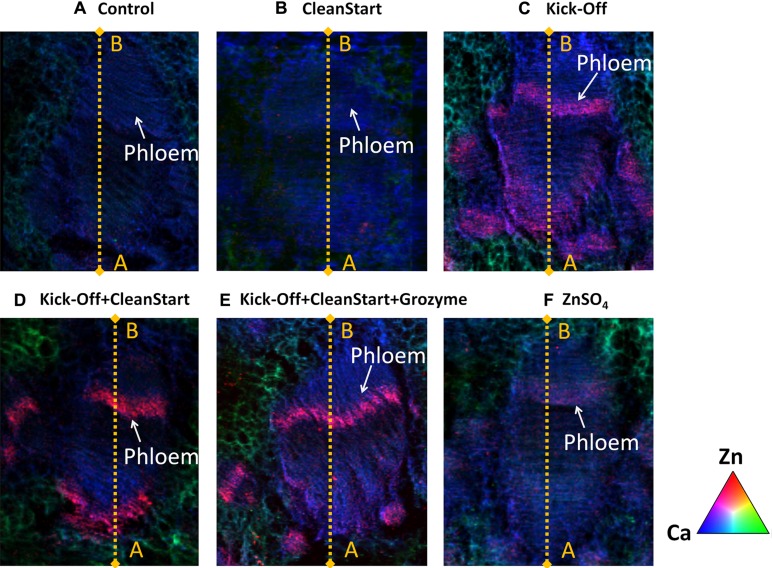
**Micro X-ray fluorescence elemental maps for Zn (red), Ca (green), and K (blue) focused on vascular bundles of petioles collected from sunflowers subject to different foliar fertilizers.** Full expanded leaves of sunflower were treated with **(A)** control, **(B)** CleanStart, **(C)** Kick-Off, **(D)** CleanStart + Kick-Off, **(E)** CleanStart + Kick-Off + GroZyme, and **(F)** ZnSO_4_. Compositions of the nutrients in different treatments were shown in **Table 1**. Pixel brightness for μ-XRF map is displayed in RGB, with the brightest spots corresponding to the highest element fluorescence.

**FIGURE 4:**
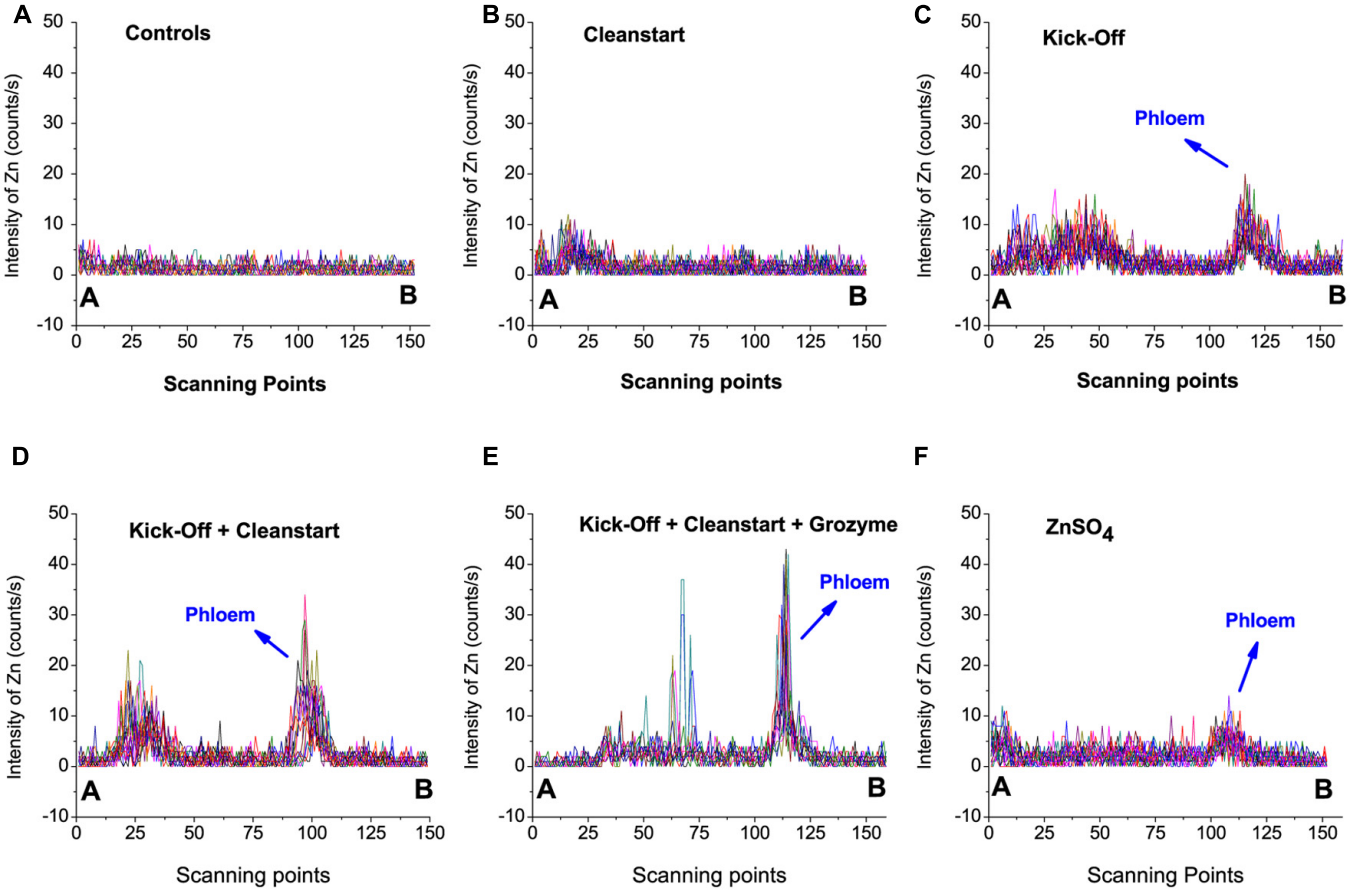
**Zinc intensities (counts/s) of 30–40 scan lines through the vascular bundles of petioles collected from sunflower plants treated with different foliar fertilizers.** The selected scanning sites from point A to B are marked by yellow lines in **Figure 3**, with 30–40 scanning different lines selected for each plant sample.

Low spatial resolution μ-XRF imaging provides only semi-quantitative data. To further investigate the effects of “CleanStart” and “GroZyme” on phloem mobility of foliar applied Zn, micro-XRF scanning at higher resolution was performed, focusing on the vascular tissues of the treated leaf veins (**Figure 3**). The Zn concentration in the petiole was also determined along a single scan line that transected the petiole and passed through the vascular system. Both “CleanStart” and “GroZyme,” which do not contain Zn, clearly increased the concentration of Zn following Kick-Off application. The patterns of Zn deposition in the “Kick-Off” + “CleanStart” and “Kick-Off” + “CleanStart” + “GroZyme” were far less diffuse and more intensely located in the phloem region and xylem/collenchyma than the pattern of Zn in petioles from leaves provided with “Kick-Off” or ZnSO_4_ alone. Intensity analysis (**Figure 4**) across a single scan line through the vascular system of the petiole demonstrated that the peak of Zn densities in the phloem tissues and xylem/collenchyma was markedly increased with addition of the biostimulant “GroZyme” to “Kick-Off” + “CleanStart” treatments (**Figure 4E**).

### TOTAL CONCENTRATIONS OF Zn IN LEAF VEINS

**FIGURE 5:**
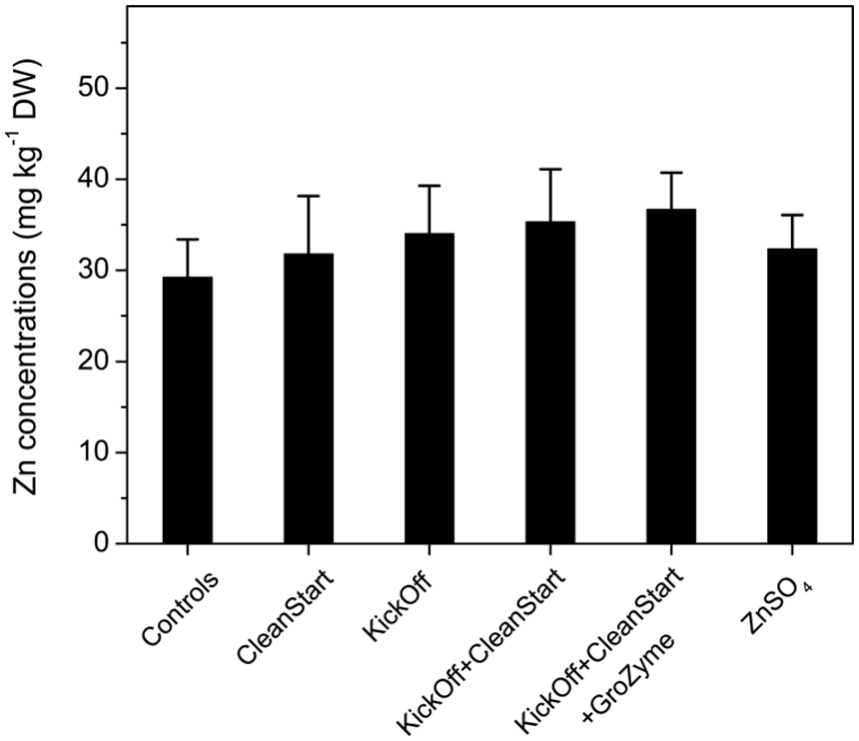
**Concentrations of Zn (mg kg^–1^ DW) in the petioles collected from sunflower plants treated with different foliar fertilizers.** Full expanded leaves of sunflower were treated with control, CleanStart, Kick-Off, CleanStart + Kick-Off, CleanStart + Kick-Off + GroZyme, and ZnSO_4_, and the Zn concentration of leaf veins were analyzed by ICP-MS. Compositions of the nutrients in different treatments were shown in **Table 1**. Data points and error bars represent means and SEs of three replicates (*n* = 3).

**FIGURE 6:**
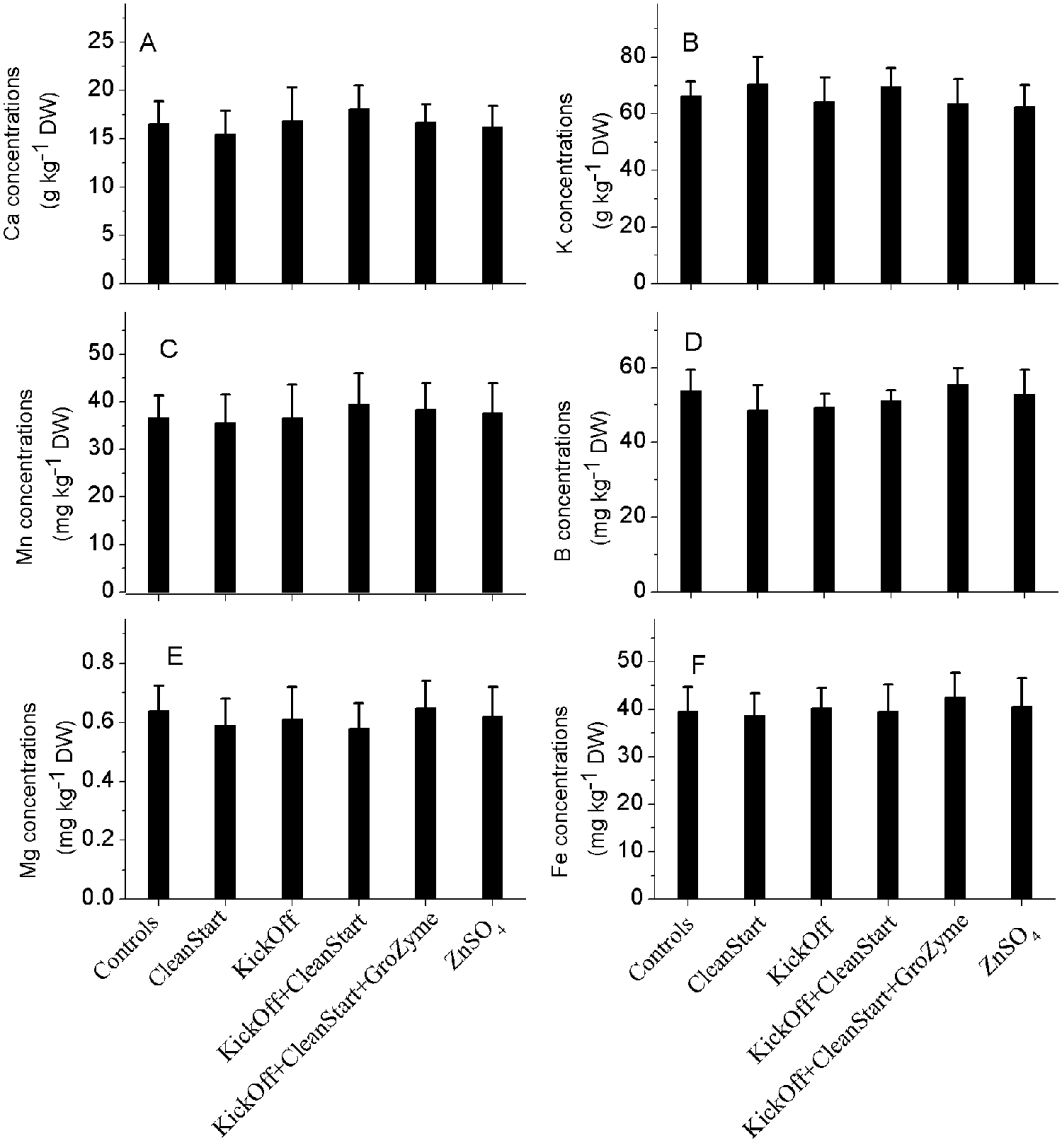
**Concentrations of Mn, Mg, B, Fe (mg kg^**–1**^ DW), and Ca, K (g kg^**–1**^ DW) in the petioles collected from sunflower plants treated with different foliar fertilizers.** Full expanded leaves of sunflower were treated with control, CleanStart, Kick-Off, CleanStart + Kick-Off, CleanStart + Kick-Off + GroZyme, and ZnSO_4_, and the concentrations of Mn, Mg, B, Fe, Ca, K of leaf veins were analyzed by ICP-MS. Compositions of the nutrients in different treatments were shown in **Table 1**. Data points and error bars represent means and SEs of three replicates (*n* = 3).

Total concentrations of Zn and other elements including Fe, K, Cu, Ca, and Mn etc. were determined by ICP-MS for the petioles collected from the sunflowers treated with different foliar fertilizers. The results showed that Zn concentrations in the leaf veins of sunflower ranged from 29.2 to 36.7 mg kg^-1^ DW. While the overall pattern of Zn concentration differences analyzed by ICPMS corresponded with the μ-XRF analysis results the total Zn concentration was not significantly different between treatments (**Figure 5**). Similarly, no difference in Fe, K, Cu, Ca, and Mn was observed among any treatments (**Figure 6**). The apparently greater sensitivity of μ-XRF is primarily a consequence of the ability of μ-XRF to analyze specifically within phloem and closely associated vascular organs while ICPMS provides analysis of the total petiole. Since vascular tissue represents only a very small proportion of the petiole as a whole, and as the petile was fully mature at the time of treatment, changes in vascular tissue element concentration may not be seen against the background of the bulk of petiole tissue in which Zn was not increased.

## DISCUSSION

Efficacy of foliar applied nutrients depends not only on the absorption of the nutrients but also on the transport of these nutrients to other plant parts (Bukovac and Wittwer, 1957; Fernández et al., 2013). It has been suggested that even a relatively small transport of foliar nutrients out of treated leaves and tissues may have a short-term, critical benefit to the plant (Fernández et al., 2013). Knowledge of the ability of an element to be transported from the site of application is critical to provide insight into the longevity and potential nutritional impact of foliar application on non-sprayed tissues.

Analysis by μ-XRF in the present study shows clearly enhanced transport and localization of Zn in the vascular system of the sunflower petiole 7 days after application of “Kick-Off,” which contains Zn-EDTA (**Figure 2C**), while Zn was not detectable in the petiole of the control plants (**Figure 2A**) and was very low in the petiole of ZnSO_4_ sprayed leaves (**Figure 2F**). This demonstrates clearly that Zn is phloem mobile in sunflower and that the use of Zn-EDTA results in greater phloem Zn transport than ZnSO_4_ alone. While it has been demonstrated that Zn-EDTA is superior to ZnSO_4_ under some circumstances, it has not been demonstrated that the EDTA molecule can penetrate the leaf cuticle. It cannot be determined from this current research if the superiority of the EDTA containing Kick-Off material is a consequence of enhanced cuticular penetration or enhanced transport of the Zn once it enters the leaf. The ‘Kick-Off’ material also contains the microelements including Fe, Cu, Mn, and Mo and this may also enhance Zn uptake as has been observed previously (Pipiska et al., 2008).

The addition of “CleanStart” derived from ammonia, urea, orthophosphoric acid and potassium hydroxide, significantly increased the phloem transport and xylem/collenchyma deposition of Zn when co-applied with ‘Kick-Off’ (**Figures 3D** and **4D**). Addition of Urea to foliar Zn sprays, for example, is known to enhance Zn uptake and efficacy (Stover et al., 1999; Johnson and Amdris, 2001; Sanchez et al., 2006) and the N status of cereals is known promote Zn retranslocation (Erenoglu et al., 2011; Barunawati et al., 2013; Xue et al., 2014). While it is clear that addition of the multi-elements present in the CleanStart enhanced Zn retranslocation into sunflower petioles, the mechanism of this effect remains uncertain.

The chemical form in which a foliar nutrient is applied will influence plant nutrient uptake by altering the point of deliquescence of the applied foliar fertilizer thereby altering its solubility on the leaf surface, or by altering the charge on the ion of interest to facilitate its movement through the cuticle and cell wall (Fernández et al., 2013). There is no direct evidence, however, to suggest that the formulation of a fertilizer spray can directly influence the transport of the absorbed nutrient from the site of application (Fernández and Brown, 2013).

The addition of the biostimulant “GroZyme” clearly enhanced Zn translocation when co-applied with “Kick-Off” and “CleanStart” (**Figure 4E**). Grozyme is a non-living microbial fermentation product derived from a proprietary mix of organic cereal grains inoculated with specific bacterial cultures and fermented. The specific functional metabolite in GroZyme has not been identified. However, extensive field trials and research published in this issue (Saa et al, this issue and unpublished research) have demonstrated positive growth effects and enhanced translocation of K and other nutrient elements (Saa et al, this issue and unpublished research). Previous research utilizing soil applications of GroZyme has also shown that this product was able to alter microbial populations in a soil environment and improve N mobilization and uptake of soil nutrients especially organic nitrogen (Chen et al., 2002, 2003). The benefit of bacterial source metabolites on efficacy of foliar fertilizers has been demonstrated previously (Ebrahim and Aly, 2004; Radzki et al., 2013) and it is plausible that the microbial extracts present in GroZyme have the capability to form metal complexes that enhance Zn uptake or mobility. Many putative biostimulants also contain plant growth hormones or plant signaling molecules that may alter plant metabolic processes and stimulate growth and indirectly influence the movement of substrates, including minerals, within the plant (Calvo et al., 2014).

In these experiment, the high spatial resolution and direct imaging capability of μ-XRF was useful in distinguishing differences in Zn transport through the vascular system of sunflower that could not be detected by ICP-MS. XRF provides a powerful strategy to trace foliar applied microelements within the plants with high sensitivity, a result that is consistent with our previous studies (Lu et al., 2013b). This technique will be useful to facilitate the development of foliar fertilizers and application techniques that optimize transport of nutrients from site of application, which is one of the most important challenges to the foliar fertilizer industry.

## Conflict of Interest Statement

The authors declare that the research was conducted in the absence of any commercial or financial relationships that could be construed as a potential conflict of interest.

## ABBREVIATIONS

μ-XRF: micro X-ray fluorescence
